# Of Conjoined Suppuration of the Gums and Alveoli

**Published:** 1842-03

**Authors:** H. H. Hayden

**Affiliations:** Baltimore.


THE AMERICAN

### JOURNAL AND LIBRARY

OF

### Dental Science.

Vol. II.] MARCH, 1842. [No. 3.

*To the Editors of the American Journal and Library of Dental Science.*

Sirs:—As a disposition has been manifested by the publishers of the American Journal of Dental Science, that the memoir written by myself, "On Conjoined Suppuration of the Gums and Alveolar Processes," and published in the Medical Recorder, of 1822, should be transferred to the pages of the Journal, you have my entire consent upon the following considerations:—

I should be sorry that the readers of that or any other journal should suppose that I am desirous for its republication on my own account, or with a view to give it a wider circulation, nor indeed, would I readily consent to its republication, but for the following reasons, viz:

It is well known that Mr. L. Koecker, an experienced dental surgeon, now of London, wrote and published in "Chapman's Journal," during his residence in Philadelphia, about the year 1819, a memoir on the "Devastation (or Conjoined Suppuration) of the Gums and Alveolar Processes," in which a case was described that indirectly implicated my professional character, or, at least, it was so understood by very competent judges, even before I saw or knew any thing of it.

Feeling a desire that the public, and more especially my professional brethren, should be in possession of correct views of the character, nature, and extent of the disease above mentioned, I

wrote the memoir alluded to. Here I could have *wished* that the matter should have remained. But inasmuch as Mr. Koecker, reiterated the same case, and in the same language, in a work which he published in London, entitled "Principles of Dental Surgery," &c. without the *slightest alteration*, or even an *allusion* to the piece I had written on the same subject—leaving me in the estimation of all those who had read only one side of the question, precisely where his first communication left me. In order, however, that the subject may be placed in its proper light, and that the public, or the profession at least, may enjoy the advantages, (if such there be), to be derived from either, I hereby assent to the re-insertion of my essay in the Journal of Dental Science, on the condition that the communication of Mr. Koecker *shall precede*, or be published in the same volume of the Journal in which my own shall be inserted.

Yours truly, H. H. HAYDEN, *Dental Surgeon.*

No. 1 North Charles-st.

### ARTICLE I.

### *Of Conjoined Suppuration of the Gums and Alveoli.* By H. H. HAYDEN, M. D., of Baltimore.

AMONG the various diseases incident to the mouth and teeth, there are but few, perhaps, that are more serious in their nature, or afflicting in their consequences, than that of a conjoined suppuration of the gums and alveoli.

This complaint, though but little known and less understood among us, has been long prevalent among the civilized inhabitants of this country as well as with those of almost every other. It appears very certain that it was even known among the ancients, but was generally confounded with what (as Mr. Hunter says) was "vulgarly called scurvy," and, as it was found to be a disease peculiar to the mouth and teeth, was called stomacace. Even Bachstrom employs the same term in his remarks and observations on the scurvy as it relates to the mouth, and the results of this disease are such as to justify the conclusion that, if not in reality, it was very nearly allied to the scurvy, though in its characters there is an essential difference.

In this light the subject was viewed, it is believed, until about the commencement of the seventeenth century, when Pierre Fauchard, an eminent dentist of Paris, wrote and published a work on the diseases of the mouth and teeth, and in which this complaint, for the first time, was treated as a distinct disease, and of which he thus observes:—"There is, moreover, another species of scurvy of which I believe no author has, as yet, taken care to speak of; and which without interesting the other parts of the body, attacks the gums, the alveoli, and teeth. Not only the gums which are soft, livid, elongated and inflated, are subject to it; but those in which none of those affections prevail are exempt from this disease."

In 1754, it is again described by Lecluse, dentist to the king of Poland, in his "Elemens d'Odontologie," p. 210.

In 1757, this disease was very accurately treated of by Bourdet, in a work entitled "l'Art du Dentiste," tom. i. p. 276, and is called "Suppuration of the Gums."

In 1771, it is again described by Auzébi, dentist at Lyons, "Traité d'Odontotechnie," p. 147. And also by Courtois and Ricci, the latter a dentist at Rheims, see "Principes d'Odontotechnie," p. 44.

In 1773, we likewise find it spoken of in an anonymous work published at Geneva, "Nouveau Traité d'Odontologie," p. 51-2.

In 1778, this truly afflicting complaint is perfectly described and treated of by Jourdain, "Maladies de la Bouche," vol. i. p. 376 to 440. In this instance the disease is termed the conjoined suppuration of the gums and alveoli.

In the same year Mr. J. Hunter published his work on "The Natural History of the Teeth," in which he describes, according to his views, the diseases of the alveolar processes, and among which the effects of this disease are mentioned; but without seeming to have any correct views of the real nature and character of the disease, although it had been so often described by others.

In 1806, Mr. Fox published his work, on the Natural History of the Teeth, and their diseases, in two quarto volumes; and in which he has attempted to describe the disease under consideration, as one of the complaints to which the alveolar processes are liable. But, although he has described the symptoms and the general appearance of the surrounding parts during the prevalence of the disease pretty accurately, it would seem that he had but a very imperfect knowledge indeed of its real character, or of the causes by which it is produced, although, as before, so much has been said of it by preceding writers.

He says "the most common disease to which they (the alveolar processes) are subject, is a gradual absorption of their substance, whereby the teeth lose their support, become weak, and at length are so loosened as to drop out. This disease usually begins to show itself between forty and fifty years of age; and from its frequent occurrence without *any evident cause,* it would seem to be a consequence of having passed the middle period of life." Fox on the Teeth, part ii. p. 87.

The disease, it must be acknowledged, is much too common, but as much so as it is, the presumption is that not more than one person in a hundred is subject to it under fifty years of age; whereas, if it was "a consequence of having passed the middle period of life," we have reason to believe that it would be much more prevalent. From the time at which Fauchard wrote upon this disease, and treated of it as being peculiar to the teeth and alveolar processes, and distinct from other diseases of the mouth, most writers have considered it as a scurvy; and it is still viewed as such by many, even at this day. Nay, so little is known and understood of its character at the present time, that there are many, and among them those who have the assurance to claim the *highest* pretensions as professional men, who have the audacity to promise that they will reinstate the teeth firm in their sockets be they ever so loose, and eradicate the disease from the gums however long they may have been diseased, and all this too under an impression that the complaint is nothing more than a simple scorbutic affection. But there is no circumstance that I have ever been able to discover, that would justify the conclusion that it has any connection with or relation to the scurvy.

Fauchard called it a species of scurvy, but one which no author had, at that time, taken care to speak of. Jourdain likewise considered it a species of scurvy, or an affection analagous to it; and at page 401, vol. ii. a genus of scurvy, as yet unknown. The circumstance that inclined him to view it as such was that the humour or matter that was discharged during the prevalence of the disease, changed the syrup of violets red.

Courtois, who was well acquainted with the disease, and has described its effects, has evidently confounded it with the scurvy.—Dentiste Observateur, p. 94.

Ricci, who has given a very concise account of the scurvy, does not mention the word in his description of the disease under consideration. Hence we are left to conclude that he considered it as distinct from that of any other.

Bourdet, whose researches and observations had been carried to an extent but seldom equalled, and which discover an intimate knowledge of the physiology and pathology of the human mouth, says, "that the suppuration of the gums does not proceed from any scorbutic taint." Hence we are left to conclude that it is an affection peculiar in its nature, and which considering the character and results of its operations, could not have a more appropriate or suitable title than that of a conjoined suppuration of the gums and alveoli.

In the next place I shall proceed to notice briefly, the several opinions that have been offered on the appearances of this disease, of the cause or causes of the symptoms, and of the effects.

Although Fauchard had observed this disease to possess characters specifically different from those of the scurvy, and although Bunon had instituted, in the Hospital de la Salpetrière and St. Come, an investigation, in order to ascertain the nature and variety of diseases of the mouth and their effects on the teeth, as well as for other purposes relating to the profession; and moreover, although Bourdet, and Tenon, had likewise been engaged in a similar investigation in one of the hospitals of Paris, with the same view and for the same purposes, the disease has been almost uniformly considered as allied to the scurvy. Hence almost every writer that has taken notice of it, has identified it with that disease, although the causes, symptoms, and results in a certain degree, but more particularly the name given it by Bourdet and Jourdain, stamp it with a character entirely different.

Of the symptoms that characterize a conjoined suppuration of the gums and alveoli, Fauchard observes, "we recognize it by a soft, livid, spongy state of the gums, and by a white and slightly glutinous pus, which is discharged from under the gums, on pressing the finger lightly from below upwards on those of the inferior jaw, and from above downwards on the superior jaw. The pus is often discharged from between the gums and the alveoli, and sometimes from between the alveolus and the root of the tooth. This occurs more frequently upon the anterior surface of the jaw and teeth, than upon the interior surface, (or part next the tongue) and more commonly about the incisors and canini of the inferior jaw than those of the superior, which are, nevertheless, more frequently subject to this complaint than the molares."

To the symptoms described by Fauchard, others are mentioned as a soft, livid and swollen state of the gums, mostly brown or lead coloured.—Bourdet. A discharge, from between the teeth and gums, of a whitish, putrid mucilage or pus, of an offensive smell.—Jourdain. Other less prominent symptoms are mentioned by several, but which it is not necessary to notice in the present instance.

Of the causes which have hitherto been considered as operating either directly or indirectly in producing this disease, most of the authors who have written on this subject, at least on the continent of Europe, and who, it is believed, are pretty generally, advocates for the humoral pathology, have assigned, among others, the following, viz: A depraved or vitiated state of the circulating fluids, in the part affected, thereby rupturing the capillary vessels and causing numerous little ulcers, which were actually found to exist by Bourdet and Tenon, on the surface of the gums next to the teeth, whence comes a purulent discharge from under the gums, so common in this disease. Hence too, it was considered by Bourdet, an erysipelatous affection. Among the remote causes are, "great exercise of mind, melancholy, bad diet, suppression of the hemorrhoides, the sudden closing up or healing, of setons, issues, &c. The sudden checks or 'repercussion' of some prevailing cutaneous disease; the putrid miasma of low and humid places, of hospitals, and also the gasseous emanations from mines." See Ricci, p. 44.

With women, the remote causes are said to be "repeated and premature suppression of the milk, or, in other words, women who do not nurse or nourish their infants from their own breasts.

Such persons as are very subject to menstrual obstructions, and also such as have experienced a total suspension of the periodical écoulement. Full and plethoric habits of body, unrestrained habits or exercises in eating and drinking, &c. &c.

Mr. Fox, and the author of an anonymous work printed at Geneva, seem disposed to attribute the cause of this disease exclusively to an accumulation of tartar upon the roots of the teeth under the gums. Of this I shall offer some remarks in the sequel.

Having given a brief exposé of this disease, and of the various opinions entertained of its character, I shall proceed to an explanation of my own views of the subject. In doing this, although I am free to acknowledge the obligations I am under to the several authors above named, (with the exception however of Messrs. Hunter and Fox,) for the light which they have shed upon this as well as on various other subjects, yet I shall not be influenced by their remarks and observations any farther than they correspond with the various appearances and indications which I have been a witness to, in numerous instances in which this complaint was committing its ravages on the human jaw and surrounding parts.

The disease in question, though various in its character, is specific in its nature—peculiar in its operations and results—and, in all cases, *primarily seated* in the investing membranes or periosteum that surrounds the roots of the teeth, and lines the respective cavities of the alveoli.

It has been, as before mentioned, generally considered as one disease, embracing almost all the affections of the gums. Hence it is that the scurvy, so called, has been confounded with it. Even those who have taken a different view of the subject, as Jourdain, Bourdet, Ricci and others, and who have denominated it, the conjoined suppuration of the alveoli and gums, have not been careful to explain *all* the different characters which it assumes, and have therefore treated it as one disease, and under one particular head. But the fact is, that although the same in its origin, and nearly the same in its results, it assumes three distinct grades, or forms, and in a manner similar to that of suppuration, caries, and partial necrosis, from an inflammation in the investing membrane of a tooth, or, in other words, a chronic tooth-ache.

The first of these grades is that which is frequently seen to attack one or more of the incisors of the inferior jaw, less frequently the four canini and the incisori of the superior jaw. But no tooth in the human mouth is exempt neither the bicuspidi nor molares. The first signs of this affection are manifested by a bright circumscribed redness about the *edge* of the gums, mostly upon the anterior surface of the tooth, and generally confined to one, but sometimes embracing two, and in a more advanced stage several teeth.

In this stage of the disease but little or no attention is paid to it. It continues to advance, until the gum is found to be detached from the tooth, for some distance in the direction of the root; but still, as the gum does not waste away as fast as the periosteum and external plate of the alveolus is destroyed, it excites little or no alarm, and is mostly neglected. Thus it is left to itself, and in proportion as it advances, the greater is the disposition in the gum to bleed on the slightest touch ; at the same time, but little or no appearance of pus is seen to be discharged ; on the contrary, a thin aqueous or slightly serous humor. Finally, if left to itself, the whole anterior plate of the alveolus, with the corresponding portion of the periosteum of the tooth, is destroyed, and the termination of the root or fang completely exposed to view, or surrounded by a portion of the inflamed lip or cheek which it is constantly excoriating.

This complaint will sometimes manifest itself on one or more teeth, and on the inner side next the roof of the mouth, but most frequently upon the molares. It gradually extends itself along by the side of the arch of the mouth, and continues its operations until the internal roots of the molares are completely exposed to view.

In this state they often remain through life, pretty firm in their sockets, though depending on the adhesions of the remaining portion of the periosteum and socket of the tooth.

If, however, it is entirely neglected, and the disease aggravated by a variety of circumstances arising from a total neglect of the mouth, the tooth or teeth will at last lose all their support, and fall out as sound as when first formed.

This is an affection to which children, as well as persons of a more advanced age, are liable; and it is believed that Mr. Fox had an allusion to it, when he speaks of persons being subject to the disease (suppuration of the gums) much earlier in life than the usual period at which it commences.

It is, moreover, a complaint which some persons do not hesitate to attempt to cure, with positive assurances at the same time that they will reinstate the gums in their former natural and healthy condition and adhering firmly to the teeth. But such an assurance and such promises betray one of two things, both alike deserving of censure. The first is, it exposes the most palpable ignorance of the physiology of the parts, and the diseases to which they are liable; or 2dly, it betrays a want of principle, or a disposition to defraud, by holding out encouragement of success in a case where every human effort must be unavailing; for it is impossible to reinstate the parts in their original condition.

In this grade of the disease as well as the third, yet to be mentioned, there is much reason to believe that it is, in some instances, hereditary, as I have often seen it prevailing with one or both of the parents, and on the *same* class of teeth in one or more of the children. It is, however, subject to control, or to be kept in check by seasonable and judicious treatment.

The second grade of the disease, though peculiar to and originating primarily in the periosteum of the teeth, like the first, differs materially in some of its characters, being generally more rapid in its progress, and far more serious in its consequences; and, moreover, being in its nature *beyond the limits* of *control*, except by the removal of all the teeth which may be involved in the disease.

The first intimation we have of this grade of the disease, is that of a dull gnawing and uneasy sensation about the part affected, without any apparent indication of disease. This is by reason of the inflammation being seated in the periosteum of the socket and surrounding the tooth. It does not, however, remain long in this state, for, if on the anterior surface of the teeth, the external plate being thin, is very soon destroyed, and the disease is communicated to the gums. It continues to advance, sometimes rapidly, until the periosteum of the tooth or teeth is completely denuded upon half or two-thirds of the length of the roots from their points.

The processes of the teeth, or so much of the external, and sometimes internal, plates of the alveoli, as are involved in the disease, are wasted away or likewise destroyed.

The gums assume a very diseased appearance, throwing out upon their surfaces morbid granulations, as in most cases of diseased bones; whence is discharged a thin ichor, which, when absorbed, produces such a degree of excitement in the lymphatics, as that they may be traced up the side of the face in a line almost as hard as a cord. The maxillary and cervical glands are, at the same time, sensibly enlarged.

From the commencement, and during the progress of this disease, the gums remain united to the neck of the tooth or teeth as usual, with a portion of the orifice of the alveolus still embracing the tooth, so that on striking the crown or body of the tooth with an instrument, and at the same time applying the finger on the gums over the tooth, a sensible motion in the root is felt, showing not only that the socket of the tooth is partially destroyed, but that the disease originated in it.

Should the disease be situated upon one or more teeth where the surrounding sockets are somewhat thick and dense, and in consequence of which it is not so readily or rapidly destroyed, the diseased periosteum will throw out, from between the gums and teeth, morbid excrescences of a luxuriant growth, and in number proportioned to the extent of the disease.

If it should attack the bicuspides and molares of the superior jaw, where, in general, the external alveolar plate is thin, morbid granulations are thrown out immediately where the bone is destroyed, and they continue to spread, in proportion to the loss of bone, and at the same time, discharging as has been mentioned, an extremely corrosive ichor.

If we attempt to remove those fungous excrescences, thrown out from between the teeth and the gums, we find the hemorrhage profuse and troublesome; and this is not the most unfavourable circumstance attending it, for their growth appears to be greatly promoted and their malignity increased. This being the case, the disease may be said to be without the limits of control, except by the extraction of the teeth affected, when the roots will be found more or less completely deprived of the investing membrane, and when this is the case, no human effort can restore it again as in other diseased bones, and for very obvious reasons.

This form of the disease I have seen in several instances, and in one as early as at fourteen years of age; and, moreover, it may always be considered as an unequivocal mark of a scrofulous habit or taint in the constitution of the patient.

The third and last grade of the disease, or that which is very appropriately called the conjoined suppuration of the alveoli and gums, though differing in some of its characters, is the same in its origin, and nearly the same in its results. The persons, seemingly, most liable to it, are those who otherwise enjoy the best of health and the soundest teeth.

The first appearance of the disease is, that the gums upon the anterior surface of one or more teeth, or upon the side next to the tongue or roof of the mouth, or even between the teeth, discover a preternatural circumscribed redness. In a more advanced state it becomes purple and even livid; somewhat swollen, and of a shining appearance. On pressing the finger, or instrument, slightly upon the diseased part, a thick purulent matter, or pus, is seen to discharge at the neck of the tooth. As the disease advances, the gums become more swollen, turgid, and of a deeper red or purple colour; while the ill conditioned pus increases in quantity and acquires, at times, a very fetid smell. In some cases it will commence upon one tooth, skip or pass by the adjoining one and attack the third, and so on all around the mouth. But commence where it may, if no attention is paid to arrest or check its progress, by the time that the socket, in which the disease begins, is so far destroyed that its respective tooth has no longer any support, the disease has begun its operations on the socket of the adjoining tooth, and so on until the whole are swept away, and nothing left to assist in mastication, but the naked gums.

It becomes necessary in the next place to say something of the cause of this complaint.

I have observed that it is primarily seated in the periosteum of the teeth, and that of the corresponding alveoli—hence a disease peculiar to these parts, and to which the periosteum of the bones of other parts are by no means exempt. Hence too, whatever tends directly to produce an irritation or inflammation in those membranes, may be considered as a proximate cause by which the disease is produced.

In the present instance, we find it arising from two causes; the one external, the other internal.

That arising from an external cause is produced by an accumulation of tartar; which, from neglect or inattention, is suffered to collect in such quantities as by insinuating itself between the gums and teeth, to press upon the periosteum, and being sharp and uneven in its surface, it thereby produces irritation, inflammation, and a partial suppuration, which if neglected, deprives the tooth of support and they ultimately fall out, though, perhaps, perfectly sound.

In this instance, three circumstances present themselves as worthy of particular notice, as it respects the relation or connection existing between the disease arising from this cause and the one yet to be described.

1st. The disease is, by no means partial in its operation. Whenever the tartar accumulates in a quantity sufficient to press upon and irritate the investing membrane, whether on the molares of the superior jaw opposite the parotid duct, or the teeth opposite, or in front of the sublingual ducts, (taking it for granted that the tartar is deposited from the saliva) the effect is the same: on pressing slightly on the gums, pus is seen to flow out from between the gums and the tartar, or from under the gums and beneath the tartar; and that too, oftentimes, from *all* the teeth on which tartar may have collected in any quantity.

This is seldom or ever the case when the disease arises from an internal cause, for it commences upon one or more *alternately*, and so on through the whole circle of the jaw.

2dly. From the slow progress of the disease in this instance, although prevailing throughout the entire circle of both superior and inferior jaws, little or no fetor is observable in the matter thus discharged, unless the disease is far advanced; nor, in fact, from any other source, except such as may arise from a want of due attention to cleanliness in the mouth, in which case, it differs essentially from that which is emitted from pus discharged from carious or otherwise diseased bones. The case is directly the reverse when the disease is produced from an internal cause.

3dly. The disease, when arising from this cause, is susceptible of perfect control, provided the teeth have not lost their support by the destruction of the greatest portion of their respective alveoli. Nothing more is necessary than a judicious and careful removal of the existing cause, the tartar—a proper attention to cleanliness, with a view to preserve the teeth free from extraneous deposites, and the use of astringent and tonic gargles, elixirs, &c. of which there are many, and such as are very efficacious. How far this disease is susceptible of control, when arising from a different cause, may be seen by the various opinions that have been advanced on the subject, and the methods that have been hitherto pursued by those who have undertaken the treatment of it.

Conjoined suppuration of the gums and alveoli, appears to be occasioned by a morbid excitement in the periosteum of the teeth and alveoli, from some internal cause, and from the period of life at which it generally attacks the patient, it has been considered by most writers on the subject, as an indication of some change in the economy of the system, or constitutional depravity in the circulating fluids by which a change in the vascular action of the part affected is produced, and which terminates in a conjoined suppuration, &c.

Hence, it has been alleged, that those who are most frequently attacked by the disease are such as are of full habit of body, or, in other words, corpulent; and those who, after the meridian of life are subject to a habitual costiveness. It is said to be also occasioned by great exercise of mind, sedentary habits, melancholy, bad diet, suppression of the hemorrhoides, the sudden closing up or healing of issues and seatons, the 'repercussion' of some prevailing cutaneous disease, &c.

With women it is said to be occasioned by the premature and repeated suppression of the milk. It is likewise considered as a consequence of obstructed catamenia, as it is found frequently to prevail with those of the sex who are "mal-reglées," or with those who have experienced a cessation of the écoulement périodique, &c.

What ever agency these circumstances may have in the production or the promotion of the disease, I shall not pretend to decide. This much, however, may be relied on—the disease, as Bourdet has very correctly observed, seldom attacks any person under thirty years of age, though I have seen three or four cases at an earlier period. From that to forty and upwards, it is much more common. Persons of corpulent habits, of either sex, are peculiarly liable to it. Women who have passed the critical period of life are, in frequent instances, afflicted with it, however good may have been their previous state of health, or that of the mouth and teeth.

And, in general, the disease, in such cases, is more rapid in its progress and more difficult of control than in almost any other, and the quantity of pus discharged is very great, although the patient is rarely sensible of it, for it is mostly mixed with the saliva and carried into the stomach, or voided by expectoration.

I have likewise observed, that persons subject to gout or rheumatism, though corpulent, are seldom afflicted with this disease. On the contrary, I have remarked that persons afflicted with a suppuration of the gums, are seldom troubled with gout or rheumatism, if so, however, the paroxysms are slight and of short duration.

Whether the disease of the gums or mouth may be considered as having any influence in preventing the gout or rheumatism, is not for me to decide in the present instance. The discharge, however, of fetid pus from under the gums during the prevalence of a "conjoined suppuration of the gums and alveoli," and that too of long continuance, may often be seen to exceed that from a seton. Hence, frequently, the extremely fetid smell accompanying this discharge of pus.

I shall next offer some brief remarks on the treatment of this complaint.

The methods hitherto pursued, in Europe, by the most skilful medical practitioners, as well as professional dentists having been so rarely successful in effecting a cure; one is left, and not without reason, to doubt the efficacy of any course of treatment which human ingenuity can invent; nevertheless, in the incipient stage of the disease, if the patient fortunately applies at that period, it is susceptible of a partial control.

At this period, as before mentioned, the gums upon the anterior surface of one or more teeth appear red, or lived and swollen. In pressing the gums slightly, a thick purulent matter is seen to discharge at the neck of the tooth.

In this, and a more advanced stage of the disease, Bourdet was in the habit of removing, by excision, the diseased portion of the gum.

This course he was led to pursue, with a view to a cure, from the following circumstance. While engaged with Tenon, in one of the hospitals in Paris, in examining the various diseases of the mouth and their effects upon the teeth, they found on dissecting up the gums in a state of suppuration, that the inside, next the teeth, was covered with little white vesicles or blisters, from which was discharged a viscid humour, which being thrown, when living, upon the periosteum and bone, was supposed to be the agent by which the alveoli are destroyed in this disease. Hence Bourdet considered it an erysipelatous affection; and finding the seat of the disease, apparently, in the flesh or gums, it was denominated a suppuration of the gums.

We are not to conclude, however, from this circumstance that the disease is primarily seated in the gums; for it will be found in many cases, that when the periosteum of a bone is attacked by disease, it presents a vesicular appearance, and from which, in certain stages, is discharged a morbid fluid. The surrounding substance assumes a similar appearance, and frequently terminates in ulceration. This is particularly the case in the mouth. Moreover, this disease often originates in the alveoli as in the second grade, and even upon the soundest tooth, commencing with an inflammation and swelling like the common paroulis, and suppuration follows. But in this instance, the matter is seldom if ever discharged through the gums: it follows the direction of the roots, and is discharged at the edge of the gums, at the neck of the tooth; and will continue so to do, though in a less degree, until the tooth or teeth thus affected are deprived of all support, and fall out, for no effort can control the disease in this case, except by extracting the tooth.

This is one instance which shows that conjoined suppuration of the gums and alveoli is primarily seated in the periosteum of the teeth and alveoli—that it is occasioned by some constitutional defect or cause—and moreover, that tartar can have no agency in the business, as it is impossible for it to gain admittance, or be formed there by the common process.

The method pursued and recommended by Bourdet I have generally adopted; not, however, that I considered that in removing the diseased gums, I likewise removed the inflamed periosteum; but that by this course the turgidity of the inflamed vessels was relieved, and the tendency to a morbid excitement checked by new and healthy granulations, supplying the place of previous disease.

By this course and the use of appropriate gargles, of which there are many, I have generally been successful in checking the progress of the disease, in some cases, even for several years. But whether as the patient advances in age, the disposition to disease in those organs increases or not, I have uniformly found the complaint to return, and, in some instances with symptoms so aggravated, and, at the same time to such an extent, as to defy all control.

This disease, as described and treated, as long ago as 1728, by the most eminent of the profession in Europe, I have been acquainted with, and in the habit of prescribing for, these twenty years past.* I have seen it in all its forms and stages, which, perhaps it ever assumes; and, although I am ready to admit, as before, that in the incipient stage it is susceptible of control, and at all times, may by proper attention and treatment, be in some degree checked, I do not hesitate to assert that a radical cure of the disease is beyond the reach of medical skill, and whoever will take a correct physiological and pathological view of the subject, will not hesitate to admit the fact.

This, however, is not to be understood as applicable to suppuration of the gums, when occasioned by the presence of any extraneous matter, as tartar upon the teeth.

If we were to admit that the complaint may be overcome and subdued, it is even said, and not without reason, that when the disease is somewhat advanced, it is even dangerous to suppress it. "Unfortunately," says Ricci, "the assistance is always required too late, and at a time when it is impossible and even dangerous to suppress this suppuration already established, and by which, nature is seeking to disembarrass her works from a morbid humour which is committing its ravages on those parts which are the most interesting, viz: the teeth.—Ricci's Principes d'Odontotechnie, p. 44.

* At this time nearly forty years.

In confirmation of this, a case is mentioned by Jourdain, in which a lady, who, otherwise enjoyed the most perfect health, was attacked by this disease, and which threatened to deprive her person of one of its greatest ornaments.

The course pursued for the treatment, was such as in a degree to check the suppuration; and while she was felicitating herself with the prospect of a speedy cure, she was, for the first time in her life, attacked with a dry cough, pains and heat in the breast, and spitting of blood, &c. Her physician, M. Bouvard, suspecting that these unpleasant symptoms were occasioned by the attempt to suppress or overcome the disease of the mouth, was under the necessity of resorting to measures calculated for the *re-establishment* of the suppuration of the gums, and which having accomplished, she was relieved from the alarming symptoms of organic disease.—Maladies de la Bouche, tom. i. p. 401–2.

Another case is mentioned by the same author, of a lady who was attacked with a conjoined suppuration of the alveoli and gums. After submitting to the usual surgical operations resorted to in similar cases, such as excision of the gums, the use of the cautery, &c. she resorted to the baths, to detergent drinks, to blisters, and even to a seton between the shoulders, without deriving any real success. Numbers of her teeth fell out of her mouth, and the others announced a disposition to follow, sooner or later.—*Ib.* p. 400.

This disease, from the nature and extent of its ravages, and these too as great or more so amongst the opulent and rich, as among the poorer classes of society, has, at different times, engaged the attention of some of the most skilful physicians, as well as professional dentists in Europe; and in the course of treatment which they have pursued, they have, severally, resorted to every means for its cure that medical skill could suggest; such as emollient, astringent and detergent gargles of various kinds; astringent, tonic, and antiseptic elixirs; mercurial washes, absorbent powders, aromatic pastes, electuaries, alteratives, sedatives, venesections, vesicatories, injections, setons, issues, excision of the diseased parts, scraping the diseased bones, repeated applications of the actual and potential cautery, &c. Notwithstanding which, their efforts have proved ineffectual.

In some cases, and, most probably, in the incipient stage of the disease; and in others, where much depended on the constitutional temperament of the patients, they have succeeded in subduing the complaint for a time only.

In their attempts at a course of internal treatment with the same view, but very little, if any better success has attended their efforts. Hençe it has been observed by Fauchard, "that which is singular, and which I have often observed, is that those who have been treated for this disease with internal medicines, such as antiscorbutics, or those of a different kind, have in no instance been cured."

It is true that there have not been wanting persons in different parts of Europe, who have widely proclaimed the virtues of pretended specifics which they have discovered, for the cure of this complaint. But their object seems to have been the profits arising from the extensive sale of their nonpareils, elixirs, &c. more than that of any intrinsic virtues which they possessed, in curing this afflicting malady. Of this, at least, they have been accused, and their charlatanic nostrums publicly ridiculed.

Such is the nature and character of the disease in question. How far the opinions which they have advanced, relative to its cure are correct, or to what extent they are to be admitted or supported, will appear by the following:

Fauchard, in his remarks on the complaint, observes, "we may conclude from what has already been said, that this disease is not to be radically cured, but when the teeth which are affected are out of the mouth," tom. i. p. 276.

Bourdet likewise remarks, (tome i. p. 187,) "if on the contrary we suffer the suppuration of the gums to become established, which indicates amongst other disorders, the destruction of the alveoli; *all the remedies which I have* mentioned afford but a very weak relief, and serve only to prolong the loss of the teeth."

In recurring to the remarks of Ricci, (Principes d'Odontotechnie, p. 44.) which I have already quoted, he further observes, "the patients, under these unfortunate circumstances, should, therefore, resolve or be reconciled to see their mouths deprived of the finest ornament. There remains for them, no other means of remedying the defect or of obviating the deformity but the usage of artificial teeth, which, under such circumstances, become indispensable to facilitate the pronunciation and mastication."

In the works of Jourdain, whose writings on this department of medical science, stand unrivalled, we find the following. "I have known many persons who have been really attacked by the disease under consideration, and which has been treated, even by the means which, it is said some have used with success, and I can protest that I have not, as yet, seen *one only that has been cured.*"—Maladies de la Bouche, tome ii. p. 399.

Hence, whatever encouragement may be held out, or promises given for the cure of this disease when it really prevails, it will be found at last that every means and every effort will prove ineffectual.

